# Berberine mitigates intracerebral hemorrhage-induced neuroinflammation in a gut microbiota-dependent manner in mice

**DOI:** 10.18632/aging.204642

**Published:** 2023-04-07

**Authors:** Jing Liu, Haotian Chen, Ting Yu, Xiongjie Fu, Cong Qian, Xiuqin Feng

**Affiliations:** 1 Department of Nursing, the Second Affiliated Hospital, School of Medicine, Zhejiang University, Hangzhou 310016, Zhejiang, China; 2 Department of Neurosurgery, Tiantai People’s Hospital of Zhejiang Province, Taizhou 317299, Zhejiang, China; 3 Department of Neurosurgery, the Second Affiliated Hospital, School of Medicine, Zhejiang University, Hangzhou 310016, Zhejiang, China

**Keywords:** intracerebral hemorrhage, berberine, microglia/macrophage, gut microbiota, neuroinflammation

## Abstract

Background: Neuroinflammation is a frequent cause of brain damage after intracerebral hemorrhage (ICH). Gut microbiota are reported to regulate neuroinflammation. Berberine has been found to have anti-inflammatory actions, including in the central nervous system. However, it is not known whether berberine regulates neuroinflammation after ICH, nor is the relationship between the antineuroinflammatory actions of berberine and the gut microbiota after ICH understood.

Methods: ICH was induced in male mice by collagenase injection. Immunofluorescent staining and quantitative real-time polymerase chain reaction (qRT-PCR) were performed to detect microglia/macrophage phenotypes. Immunofluorescent staining, ELISA, and FITC-dextran were conducted to determine gut function. 16S rRNA sequencing of the fecal material was conducted to determine alterations in the gut microbiota. Antibiotic cocktail treatment and fecal microbiota transplantation (FMT) were used to deplete or restore the gut microbiota, respectively. Cylinder, forelimb placement and wire hanging tests were conducted to evaluate neurobehavioral function.

Results: Berberine significantly reduced neuroinflammation and alleviated neurological dysfunction by preventing microglial/macrophage proinflammatory polarization in ICH mice. Berberine also enhanced the function of the intestinal barrier, as shown by reductions in the levels of lipopolysaccharide-binding protein. Neuroinflammation in ICH mice was markedly reduced after transplantation of microbiota from berberine-treated mice, similar to treatment with oral berberine. In addition, a reduction in the microbiota reversed the neuroprotective effect of berberine.

Conclusions: Berberine is a potential treatment for ICH-induced neuroinflammation, and its effects are at least partially dependent on the gut microbiota.

## INTRODUCTION

Intracerebral hemorrhage (ICH) is a stroke subtype caused largely by aging and hypertension [1]. Both primary and secondary brain injuries may occur as a result. Primary injuries are caused by the presence and growth of hematomas, while secondary injuries are mediated by blood components, resulting in inflammation, oxidative stress, cytotoxicity, and cellular apoptosis [2]. While surgical removal of hematomas may reduce injury, it is not entirely successful in improving neurological function, and new treatments are urgently required [3, 4].

Microglia/macrophages in the central nervous system (CNS) belong to the innate immune system and are closely involved in neuroinflammation and thus in ICH prognosis [5]. Earlier studies have described genetic factors, cell receptors, and signaling pathways that are involved in neuroinflammation after ICH [6, 7]. Specifically, microglia can polarize to produce pro-inflammatory or anti-inflammatory markers at different times after ICH, and we have used these markers to analyze ICH progression [8]. Recent reports have implicated the gut microbiota in recovery from acute CNS injury. The microbiota is composed of trillions of microorganisms that have been found to influence the physiological functioning of the host. The so-called “gut-brain axis” has been implicated in a variety of CNS diseases, including stroke (both ischemic and hemorrhagic) and spinal cord (SCI), and traumatic brain injuries (TBI). Intestinal microorganisms have been linked to the modulation of inflammation and recovery after ischemic stroke, as well as gastrointestinal and locomotory functions and inflammation, after SCI in mice [9–12]. TBI affects the gut microbiota, impacting neurogenesis and fear memory, as well as neuroinflammation, and ICH has been observed to induce similar effects [13–15].

Berberine (Ber) is a medicinal herb that is widely used for treating inflammation-associated conditions and diabetes in traditional Chinese medicine [16]. Ber has been found to be effective in treating both inflammation and suppressing immune activity [17]. Many recent studies have reported that Ber mediates its effects through the gut microbiota [18]. Ber has also been found to attenuate damage to the blood–brain barrier and to improve neurological function [19]. However, it is unclear whether Ber can regulate neuroinflammation induced by ICH and whether such effects are dependent on the gut microbiota.

Here, we investigated the actions of Ber on neuroinflammation induced by ICH, including an investigation of microglial/macrophage phenotypes and intestinal function. Ber reduced inflammation and promoted both neurological and intestinal recovery after ICH. In terms of the mechanism underlying the action of Ber, neuroprotection and improved neurobehavioral functioning were observed after the administration of Ber-treated feces. These effects were reversed by treatment with broad-spectrum antibiotics (ABX). These findings suggested that Ber-mediated neuroprotection was dependent on the gut microbiota and provide evidence for the action of Ber in modulating the gut-brain axis, suggesting new methods of treating ICH.

## MATERIALS AND METHODS

### Animals

Mice (adult male C57, 8–10 weeks old, 23–25 g) were obtained from Charles River Laboratory Animal Co., Ltd. (Beijing, China). The animals were housed in a specific pathogen-free (SPF) environment with a 12 h/ 12 h dark/light cycle and ad libitum access to food and water, with five animals per cage. All procedures were performed according to the Guide for the Care and Use of Laboratory Animals of the National Institutes of Health and were approved by the Institutional Ethics Committee of the Second Affiliated Hospital, Zhejiang University of Medicine (animal ethic approval batch number AIRB-2002-0994).

### Study design

#### Experiment 1

To examine the effects of Ber on the microglia/ macrophage phenotypes, 28 animals were randomly allocated to three groups, and microglia/macrophage phenotypes were assessed three days after ICH after Ber treatment or no Ber treatment using immunofluorescence and qRT-PCR (Supplementary Figure 1).

#### Experiment 2

To evaluate the effects of Ber after ICH, 20 mice were allocated to two groups (*n* = 10 per group). The first group (ICH + Vehicle) received only the Vehicle used in the Ber solution, and the second group (ICH + Ber) was treated with Ber. Neurological function was assessed at one, two, and three days post-ICH.

#### Experiment 3

To determine the effects of Ber on gut function three days after ICH, 40 mice were assigned to three groups [Sham (*n* = 10), ICH + Vehicle (*n* = 15), and ICH + Ber (*n* = 15)]. The first group was evaluated by immunofluorescent staining, gut permeability was examined by plasma fluorescence in the second group, and the third group was assessed using ELISA. The functioning of the intestinal barrier was analyzed by immunofluorescence and LBP assays to determine the effects of Ber on gut function. Intestinal permeability was examined by the measurement of fluorescence in the plasma of FITC-dextran (FD-4) three days post-ICH with and without Ber administration.

#### Experiment 4

The role of the gut microbiota was investigated using ABX treatment. Three groups of 26 mice each were established: Sham, ICH + Vehicle + Ber, and ICH + ABX + Ber. The mice received fresh broad-spectrum ABX in their drinking water for four weeks before ICH, after which they were treated with Ber and evaluated by neurobehavioral assessments, immunofluorescence staining, and qRT-PCR.

To determine alterations in the gut microbiota after ICH and ABX treatment, mouse feces were collected before and after antibiotic treatment, and 16S rRNA sequencing of the fecal material was conducted.

#### Experiment 5

Fecal microbiota transfer (FMT) experiments were then conducted to examine the action of Ber. Three groups of 26 mice per group were assessed by neurobehavioral evaluation, immunofluorescence, qRT-PCR, gut permeability, and ELISA [Sham (*n* = 16), ICH + FMT (Vehicle) (*n* = 31), and ICH + FMT (Ber) (*n* = 31)].

### Mouse model

The establishment of the ICH model followed a previously described protocol [20]. Briefly, mice were anesthetized by intraperitoneal administration of pentobarbital sodium (40 mg/kg, 1%) and collagenase (0.05 U type VII collagenase in 0.5 μl saline, from Clostridium histolyticum; Sigma–Aldrich) was stereotactically injected into the right basal ganglia (2.5 mm lateral to the bregma, 3 mm deep at a 5° angle). The rectal temperature of the mice was kept constant at 37.0 ± 0.5°C. The sham-operated group was treated similarly, but no collagenase was included in the injection.

### Berberine administration

Ber (Sangon Biotech, Shanghai, China) was dissolved in 0.9% saline to 25 mg/ml as previously described [19]. Ber dose (200 mg/kg/d) was administered by oral gavage, with the vehicle-only group mice receiving an equal volume of saline. The first dose was administered 3 hours after ICH modeling and then daily until the animal was sacrificed.

### Neurobehavioral assessments

Evaluations were performed by two blinded investigators between one and three days after ICH. Three behavioral tests, namely, the cylinder, forelimb placement, and wire-hanging tests, were conducted [21, 22]. In the cylinder test, the animals were placed into a transparent cylinder (diameter: 8 cm; height: 25 cm) where they could rear. Twenty rears were assessed, and the first forepaw touch on the cylinder wall was documented. The scores were determined as (right-left)/ (right + left + both), with higher positive scores indicating greater left hemiparesis. Forelimb placing was assessed by holding the mouse and allowing the forelimbs to dangle freely. After testing each forelimb 10 times, the number of times the forelimb was placed on the countertop edge after stimulating the whiskers was noted. In the wire-hanging assessment, the mouse was positioned on a 50-cm long and 2-mm diameter bar suspended by two vertical supports 37 cm above the surface. Three trials of 30 s each were conducted, and scores were determined as follows: 0, the mouse fell off; 1, the mouse held onto the bar with both forepaws; 2, the mouse held onto the bar with attempts at climbing; 3, the mouse held onto the bar with both forepaws and at least one rear paw; 4, the mouse used all four paws and the tail to hold onto the bar; and 5, the mouse escaped down the supporting structures.

### qRT-PCR

After anesthesia and intracardiac perfusion with 0.1 M chilled PBS, the mouse brains were removed and gently dissected. Total RNA was extracted from the hematoma in the basal ganglion region with TRIzol (Invitrogen, Thermo Fisher, Waltham, MA, USA) following the provided directions and reverse-transcribed to cDNA using PrimeScript^™^ RT Master Mix (Takara BioInc, Shiga, Japan). qRT-PCR was conducted according to standard protocols using Applied Biosystems Quant Studio and TB Green Premix Ex Taq. Melting curve analysis was used to evaluate reaction specificities, and β-actin was used as a control for normalization. Gene expression was normalized to that of the control and was calculated with the 2^−ΔΔCt^ method. Primer sequences are provided in Table 1.

**Table 1:** Primers used in qRT-PCR.

**Primer sequences (5′-3′)**
**Gene**	**Forward**	**Reverse**
IL-1β	CAACCAACAAGTGATATTCTCCATG	GATCCACACTCTCCAGCTGCA
TNF-α	ATGGCCTCCCTCTCAGTTC	TTGGTGGTTTGCTACGACGTG
CD16	ATGCACACTCTGGAAGCCAA	AAGAGCACTCTGCCTGTCTG
Arg-1	CGCCTTTCTCAAAAGGACAG	CCAGCTCTTCATTGGCTTTC
CD206	CAAGGAAGGTTGGCATTTGT	CCTTTCAGTCCTTTGCAAGC
β-Actin	AGGCATTGTGATGGACTCCG	AGCTCAGTAACAGTCCGCCTA

### Immunofluorescence

Mice were perfused with 20 mL of cold 0.1 M PBS and subsequently with 4% PFA as described earlier while under deep anesthesia. The brains were removed, fixed with PFA overnight, and treated with 30% sucrose at 4°C for an additional 72 h. Ten-micrometer coronal sections were then cut for further analysis. The mouse colons were fixed with 4% PFA overnight. Sections (5 μm) were cut, washed in PBS, and blocked with 10% donkey serum with 0.3% Triton X-100 (room temperature, 1 h) before incubation with anti-Iba-1 (1:500, Abcam, ab5076), anti-CD16 (1:100, Abcam, ab25235), anti-Arg-1 (1:500, Proteintech, 16001-1-AP), and anti-claudin (1:50, Santa Cruz Biotechnology, sc-166338) antibodies (4°C, overnight). After rewashing, the sections were incubated with species-appropriate anti-IgG antibodies conjugated with Alexa Fluor 488, 555, or 594 at 37°C for 1 h and stained with DAPI (Abcam, ab104135). Three sections per animal were assessed under fluorescence microscopy (Leica, Mannheim, Germany). Target cells in brain sections were observed in three fields, the means were determined, and the fluorescence intensities in the colonic sections were analyzed by ImageJ software (NIH).

### Measurement of intestinal permeability

Mice received 0.6 g/kg FITC-dextran (0.1 mg/ml dissolved in 1xPBS; 4 kDa; Sigma) by oral gavage after a 12-h fast. Blood samples were taken four hours later and centrifuged (2500 g, 10 min) to obtain the plasma. Fluorescence levels in the plasma were examined in a fluorescence spectrophotometer (SoftMax^®^ Pro5, Molecular Devices) using excitation and emission wavelengths of 485 and 535 nm, respectively. The readings were normalized to the blanks and expressed as the fluorescence percentages for each mouse. The test was conducted by blinded investigators.

### Determination of lipopolysaccharide-binding protein (LBP) levels

LBP levels in the circulation were measured using an ELISA kit (Nanjing Jian Cheng Bioengineering Institute, China) following the provided directions. Briefly, the plasma samples were placed in the appropriate wells, and after incubation with specific antibodies, the samples were treated with an HRP-conjugated antigen for 30 min at 37°C, leading to competition between the antigen on the solid phase and the newly formed immune complex. The HRP catalyzed tetramethyl benzidine breakdown to a blue color, which was transformed to yellow upon interaction with acid. Absorbances were read at 450 nm.

### ABX treatment for germ-free mouse modeling

Broad-spectrum ABX was administered to mice in their drinking water for four weeks with renewal every other day. The ABX comprised of ampicillin (500 mg/L), metronidazole (500 mg/L), neomycin (500 mg/L), and vancomycin (250 mg/L) (Sangon Biotech, Shanghai, China) and with 5% sucrose in pure distilled water. This treatment eliminated most of the fecal bacteria, as has been shown in many studies [23, 24].

### Fecal microbiota transfer (FMT)

As previously described, feces were obtained between 09:00 and 10:00 from five ICH mice after Ber treatment and were suspended in chilled 1X PBS (120 mg feces/ 1 ml PBS) [25]. Feces from the same group of animals were collected together, after standing for five minutes, the feces were homogenized for 10 min and centrifuged (1000 × g, 10 min, 4°C). The supernatant was retained and transplanted into mice that had received broad-spectrum ABX by oral gavage for four weeks before ICH. One hundred microliters of the supernatant were administered by oral gavage to the mice after ICH daily for three days.

### Gut microbiota analysis

Sequencing of the 16S rRNA amplicon was conducted by LC-Bio Technology Company (Hangzhou, Zhejiang, China). The abdomens of the mice were massaged to obtain fresh feces to prevent exogenous bacterial contamination. DNA was isolated from the feces using an E.Z.N.A.^®^ Stool DNA Kit (D4015, Omega, Inc., USA) following the provided protocol. The DNA contents of the samples were measured with a Qubit fluorometer (Thermo Fisher, Waltham, MA, USA). The 16S rRNA sequences were amplified using V4 region-specific primers. AMPure XT beads (Beckman Coulter Genomics, Danvers, MA, USA) were used for purification, and Qubit beads were used for quantification (Invitrogen, Waltham, MA, USA). The amplicon library was evaluated using an Agilent 2100 Bioanalyzer (Agilent, Santa Clara, CA, USA) and a Library Quantification Kit for Illumina (Kapa Biosciences, Woburn, MA, USA) and sequenced on an Illumina NovaSeq PE250 platform (LC Bio). The paired-end reads were assigned according to their barcodes and were truncated by removal of the barcode and primer sequences. The reads were then merged using FLASH software. The raw reads were filtered to obtain high-quality clean reads, and chimeric sequences were filtered with Vsearch. DADA2 was used for dereplication, and a feature table and sequence were obtained. The alpha and beta diversities were determined by random normalization to the same sequences, and abundances were normalized using the SILVA classifier with abundance normalized using the relative abundances of the individual samples. Species diversity was analyzed with alpha diversity using five indices, namely, Chao 1, Observed species, Goods coverage, Shannon, and Simpson, using QIIME2. Beta diversity was determined with QIIME2, and R was used to draw graphs. Sequences were aligned in BLAST and annotated by SILVA. Additional diagrams were drawn using R V3.5.2.

### Statistical analysis

Data were shown as the mean ± standard error of the mean (SEM). T tests were used for the analysis of normally distributed data and the differences between two groups. Persistent neurological functions were analyzed by two-way repeated-measures ANOVA followed by Tukey’s post hoc test. Nonnormally distributed data were compared with Kruskal–Wallis tests and Dunn-Bonferroni tests for post hoc comparison. Statistical significance was set at *P* < 0.05. Statistical analyses were performed using GraphPad Prism 8.0 (GraphPad Prism Software Inc., San Diego, CA, USA) and SPSS 22.0 for Windows (IBM Corp., Armonk, NY, USA).

### Data availability statement

The raw data supporting the conclusions of this article will be made available by the authors, without undue reservation.

## RESULTS

### Berberine reduced neuroinflammation through modulation of the microglial phenotype after ICH

**Figure 1:**
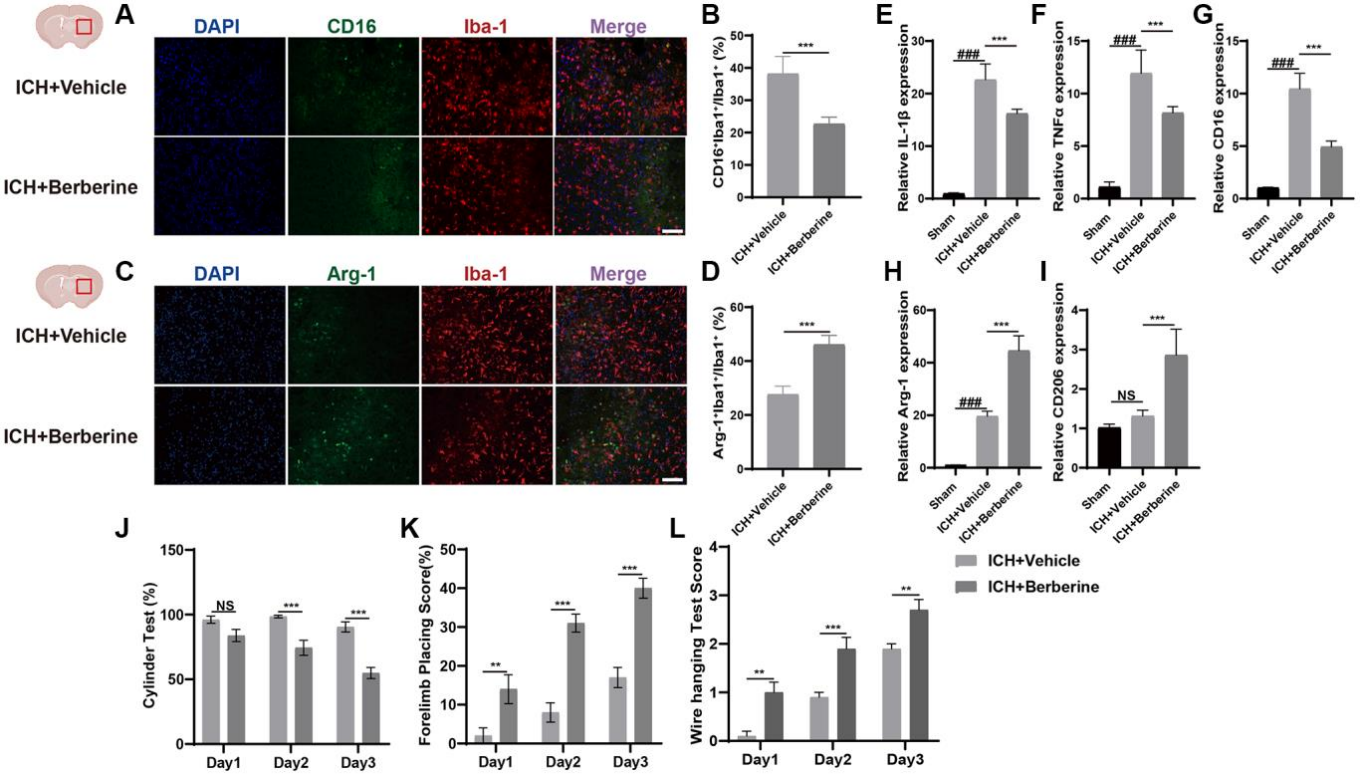
**Berberine promoted the phenotype of microglia/macrophage transformed into anti-inflammation and ameliorated ICH-induced brain injury and improves neurological function.** (**A**, **B**) Immunostaining for CD16^+^Iba-1^+^/Iba-1^+^ in ICH + Vehicle and ICH + Ber group after ICH day 3. (*n* = 5 per group). (**C**, **D**) Immunostaining for Arg-1^+^Iba-1^+^/Iba-1^+^ in ICH + Vehicle and ICH + Ber group after ICH day 3. (*n* = 5 per group). (**E**–**G**) The levels of mRNA transcription of IL-1β, TNF-α, and CD16 were examined by qRT-PCR in sham, ICH + Vehicle, and ICH + Ber groups after ICH day 3. (*n* = 6 per group). (**H**, **I**) Relative mRNA expression of Arg-1 and CD206 in sham, ICH + Vehicle, and ICH + Ber groups after ICH day 3. (*n* = 6 per group). (**J**–**L**) The percentage of cylinder test, forelimb test, and the score of wire hanging test in ICH + Vehicle and ICH + Ber group. (*n* = 10 per group). Data are expressed as the mean ± SEM. ^#^*P* < 0.05. ^##^*P* < 0.01. ^###^*P* < 0.001 sham vs. ICH + Vehicle group. ^*^P < 0.05. ^**^P < 0.01. ^***^P < 0.001 ICH + Vehicle group vs. ICH + Ber group. Scale bar = 100 μm.

Phenotypic changes in hematoma-associated microglia after Ber treatment were analyzed using immunofluorescence. The numbers of CD16^+^Iba^+^/Iba^+^ cells and the Arg-1^+^Iba^+^/Iba^+^ cell ratio are shown in Figure 1. The percentages of CD16^+^Iba-1^+^ cells were significantly reduced in Ber treated group (Figure 1A, 1B), while greater Arg-1^+^Iba^+^/Iba^+^ cell ratios were observed in the Ber-treated mice three days after ICH (Figure 1C, 1D).

Changes in inflammatory gene expression were also evaluated in the brain hemisphere after ICH. Temporal changes in the levels of inflammation-associated genes were also evaluated in the affected brain hemisphere after ICH. The mRNA levels of IL-1β, TNF-α, and CD16 (pro-inflammatory microglial factors) were significantly upregulated on the third-day post-ICH but were reduced after Ber treatment (Figure 1E–1G). Arg-1 and CD206 (anti-inflammatory microglial factors) were higher in the ICH + Ber group than in the Vehicle group (Figure 1H, 1I). These findings indicate that Ber lowered inflammation by modulating microglial phenotypes after ICH.

### Berberine ameliorated ICH-induced damage and enhanced neurological function

We then determined the effect of Ber on ICH-induced brain damage. The animals were treated with Ber or vehicle before ICH, and the effects were assessed. After administration of Ber or vehicle to ICH mice, several behavioral tests were conducted, including the cylinder, forelimb placement, and bar-hanging tests. The cylinder test indicated significant score reductions in the Ber-treated mice compared to those treated with vehicle only on the second and third days (Figure 1J). Forelimb placement assessment showed significantly improved performance in Ber-treated mice on the third day (Figure 1K). Similarly, the Ber-treated animals scored better in the bar-hanging test, showing lower scores on Days 1, 2, and 3 (Figure 1L).

### Berberine improved intestinal integrity and lowered plasma LBP after ICH

**Figure 2:**
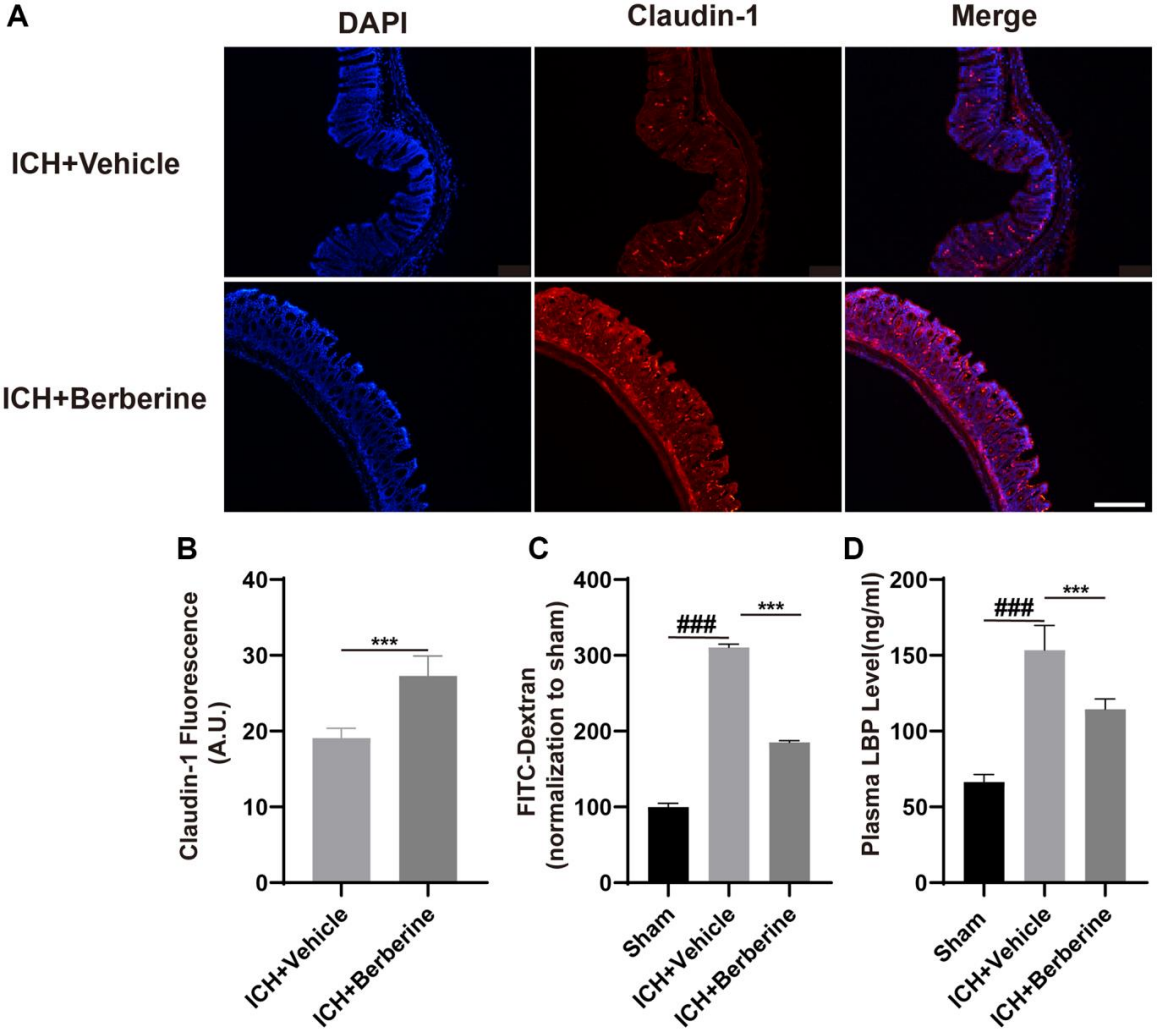
**Effect of berberine on gut permeability and gut intestinal integrity after ICH.** (**A**) The representative image of immunofluorescence staining of the tight junction protein Claudin-1 in ICH + Vehicle and ICH + Ber at day 3. (*n* = 5 per group). (**B**) The mean of densities of Claudin-1 after ICH day 3. (**C**) Ber treatment improved the intestinal permeability after ICH day 3. (*n* = 5 per group). (**D**) The level of LBP in plasma after Ber treatment day 3 in ICH mice. (*n* = 5 per group). Data are expressed as the mean ± SEM. ^#^*P* < 0.05. ^##^*P* < 0.01. ^###^*P* < 0.001 sham vs. ICH + Vehicle group. ^*^P < 0.05. ^**^P < 0.01. ^***^P < 0.001 ICH + Vehicle group vs. ICH + Ber group. Scale bar = 200 μm.

ICH is documented to increase the permeability of the mouse intestine [13]. As the levels of tight junction proteins are known to be linked to the integrity of the intestinal barrier, we measured the colonic levels of Claudin-1 after ICH. This showed that Ber treatment restored the fluorescence of Claudin-1 by the third day in comparison with the Vehicle-only group (Figure 2A, 2B). The effects of Ber were further investigated using groups of mice treated with FD-4 by oral gavage three days post-ICH, followed by measurement of FD-4 levels in the plasma. This showed that Ber could reverse the ICH-mediated damage to the intestinal barrier three days after ICH (Figure 2C). Damage to the barrier resulting in increased permeability allows the transit of bacteria and their products, such as LPS, across the intestine and into the circulation, where LPS binds to the soluble plasma protein LBP. Thus, the LBP concentration in the plasma functions as a marker for intestinal permeability. LBP levels increased significantly in ICH mice three days after ICH, while treatment with Ber reversed this increase (Figure 2D).

### Gut microbiota depletion abolished the neuroprotective actions of Ber after ICH

**Figure 3:**
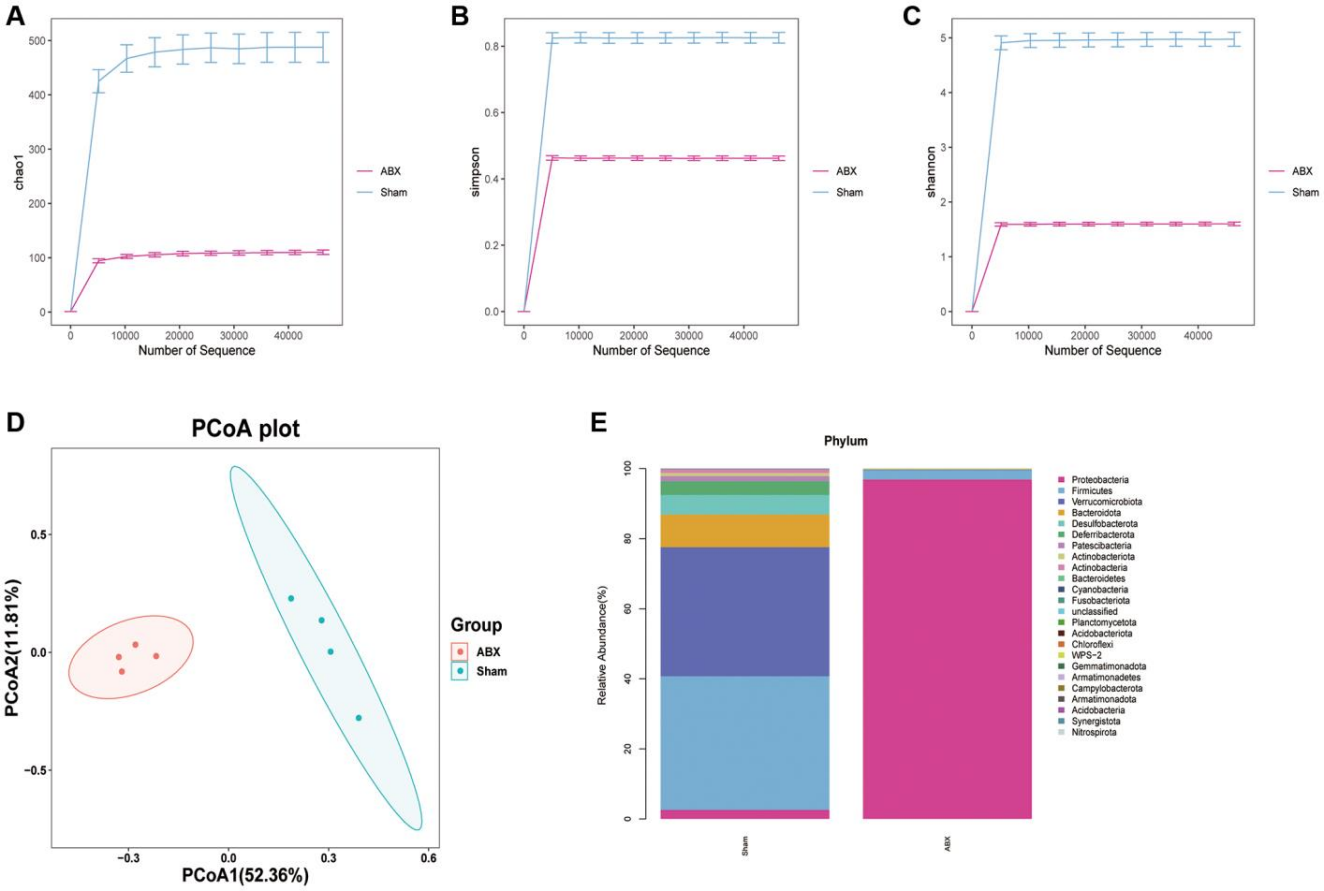
**The change of community structure of gut microbiota after ABX treatment.** (**A**–**C**) The α-diversity of gut microbiota is represented by Chao1, Simpson, and Shannon index in pre-ABX and ABX group mice. (**D**) The β-diversity of gut bacterial was represented by PCoA analysis between two groups. (**E**) The bar chart of most differentially abundant features at the Phylum level of gut microbiota in pre-ABX and ABX group mice. (*n* = 4 per group).

Three groups of mice were established, namely, Sham, ICH + Vehicle + Ber, and ICH + ABX + Ber. Changes in the microbiota after ABX administration were initially investigated using high-throughput 16S rRNA sequencing of mouse feces before and after treatment with ABX. Assessment of the alpha diversity of the gut microbiota, specifically, the Chao1, Simpson, and Shannon indices, showed that treatment with ABX resulted in a significant decrease in these indices (Figure 3A–3C). Analysis of beta diversity by principal coordinate analysis (PCoA) indicated that the community structures of the ABX-treated and untreated mice were different (Figure 3D). We then compared the composition of the gut microbiota before and after ABX administration. Reductions in various bacterial phyla, including *Firmicutes, Verrucomicrobiota, Bacteroidota, Desulfobacterota,* and *Deferribacteria,* were observed after antibiotic treatment (Figure 3E). Overall, the findings showed that ABX treatment led to significant depletion of the gut microbiota.

**Figure 4:**
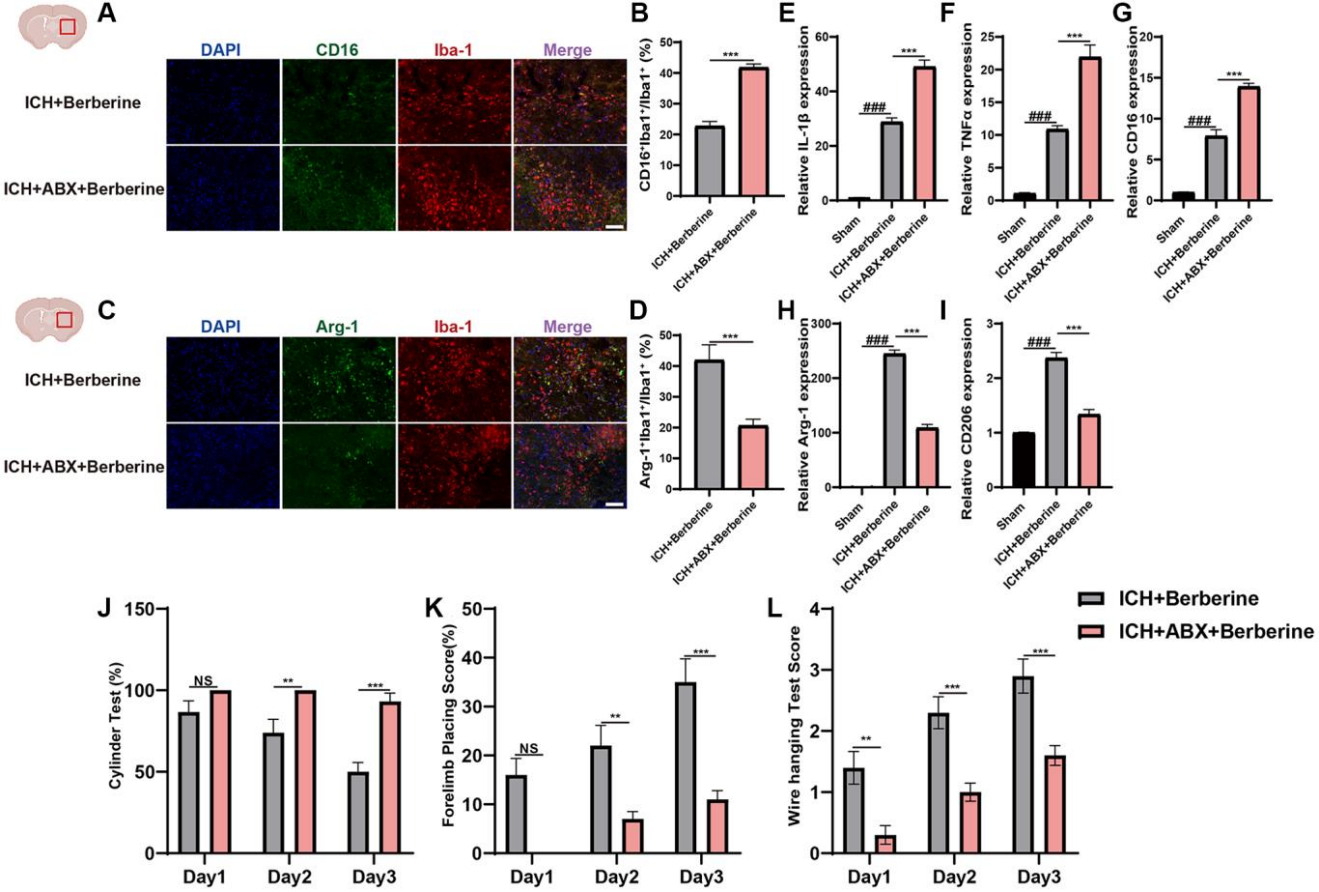
**Berberine ameliorated ICH-induced neuroinflammation and brain injury after ICH in gut microbiota-dependent manner.** (**A**, **B**) Before ICH, the mice were received the broad-spectrum ABX treatment for 4 weeks. Immunostaining for CD16^+^Iba-1^+^/ Iba-1^+^ in ICH + Vehicle + Ber and ICH + ABX + Ber group after ICH day 3. (*n* = 5 per group). (**C**, **D**) Immunostaining for Arg1^+^Iba-1^+^/Iba-1^+^ in ICH + Vehicle + Ber and ICH + ABX + Ber group after ICH day 3. (*n* = 5 per group). (**E**–**G**) qRT-PCR examined the levels of mRNA transcription of IL-1β, TNF-α, and CD16 in ICH + Vehicle + Ber and ICH + ABX + Ber group after ICH day 3. (*n* = 6 per group). (**H**, **I**) qRT-PCR examined the levels of mRNA transcription of Arg-1 and CD206 in ICH + Vehicle + Ber and ICH + ABX + Ber group after ICH day 3. (*n* = 6 per group). (**J**–**L**) Cylinder test, forelimb place test, and the wire hanging test in ICH + Vehicle + Ber and ICH + ABX + Ber group after ICH day 3. (*n* = 10 per group). Data are expressed as the mean ± SEM. ^#^*P* < 0.05. ^##^*P* < 0.01. ^###^*P* < 0.001 sham vs. ICH + Vehicle + Ber group. ^*^P < 0.05. ^**^P < 0.01. ^***^P < 0.001 ICH + Vehicle + Ber vs. ICH + ABX + Ber group. Scale bar = 100 μm.

We then investigated whether the changes in gut microbiota produced by ABX affected ICH-induced neuroinflammation. Ber treatment was found to significantly lower the proportion of CD16^+^Iba^+^/Iba^+^ cells in the ICH + Vehicle + Ber mice. However, ABX pretreatment reverses the protective effect of reduced CD16+Iba+ cells following Ber treatment (Figure 4A, 4B). Administration of ABX was also found to counteract the increased Arg-1^+^Iba^+^/Iba^+^ cell ratio in Ber-treated ICH mice (Figure 4C, 4D). Furthermore, we evaluated temporal alterations in the levels of inflammation-associated proteins in the injured brain after ABX treatment and the consequent depletion of the gut microbiota. This showed significant reductions in the mRNA levels of the inflammatory factors IL-1β, TNF-α, and CD16 in Ber-treated mice, while the opposite was seen in the ICH + ABX + Ber mice (Figure 4E–4G). An opposite trend was observed in the CD206 and Arg-1 levels between the ICH + Vehicle + Ber and ICH + ABX + Ber mice (Figure 4H, 4I), and the neuroprotective effects of Ber were not observed in the ICH + ABX + Ber group (Figure 4J–4L). Thus, these findings strongly suggest the involvement of the gut microbiota in Ber-mediated reductions in neuroinflammation and enhancement of neurological function.

### FMT from Ber-treated ICH mice reduced neuroinflammation through regulation of the microglial phenotype

**Figure 5:**
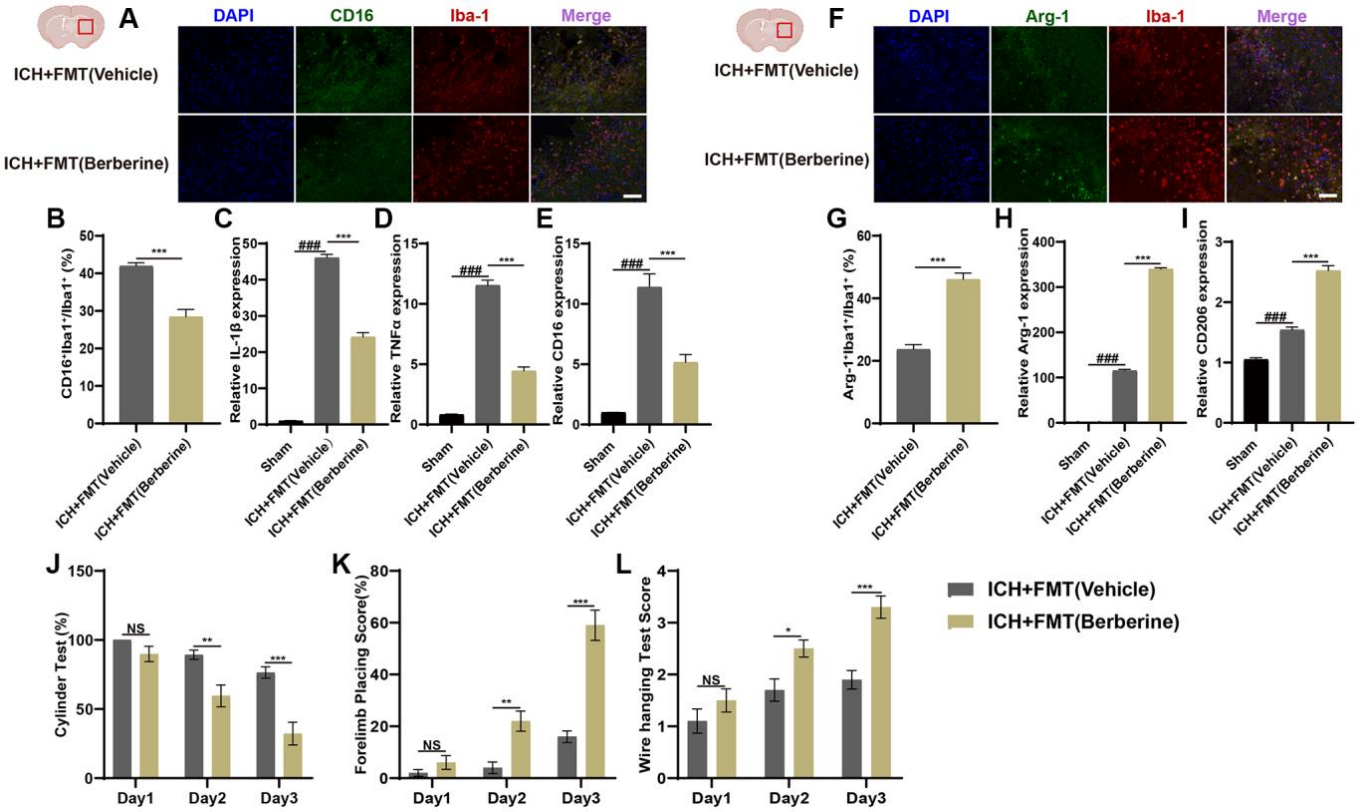
**Transplantation of fecal microbiome from berberine treatment ICH mice alleviates neuroinflammation via regulating microglia/macrophage phenotype and improves the neurobehavioral function after ICH.** (**A**, **B**) Immunostaining for CD16^+^Iba-1^+^/Iba-1^+^ in ICH +FMT (Vehicle) and ICH + FMT (Ber) group after ICH day 3. (*n* = 5 per group). (**C**–**E**) Relative mRNA expression of IL-1β, TNF-α, and CD16 in ICH + FMT (Vehicle) and ICH + FMT (Ber) groups after ICH day 3. (*n* = 6 per group). (**F**, **G**) Immunostaining for Arg-1^+^Iba-1^+^/ Iba-1^+^ in ICH + FMT (Vehicle) and ICH + FMT (Ber) group after ICH day 3. (*n* = 5 per group). (**H**, **I**) Relative mRNA expression of Arg-1 and CD206 in ICH + FMT (Vehicle) and ICH + FMT (Ber) groups after ICH day 3. (*n* = 6 per group). (**J**–**L**) Transplanted ICH mice with the fecal microbiome obtained from Ber treatment ICH donors improved the neurological function as assessed by the cylinder test, forelimb placing test, and wire hanging test after FMT (*n* = 10 per group). Data are expressed as the mean ± SEM. ^#^*P* < 0.05. ^##^*P* < 0.01. ^###^*P* < 0.001 sham vs. ICH + FMT (Vehicle) group. ^*^P < 0.05. ^**^P < 0.01. ^***^P < 0.001 ICH + FMT (Vehicle) vs. ICH + FMT (Ber) group. Scale bar = 100 μm.

The effects of FMT on microglial phenotypic changes in the hematoma region of the affected brain hemisphere were investigated by immunofluorescence. Figure 5 shows the CD16^+^Iba^+^/Iba^+^ cell and Arg-1^+^Iba^+^/Iba^+^ cell ratio results. There was a significant reduction in the percentages of CD16^+^Iba-1^+^ cells in FMT+Ber group after ICH (Figure 5A, 5B), and temporal alterations in the expression of inflammation-associated genes in the ICH-affected brains after FMT were also examined. The IL-1β, TNF-α, and CD16 levels were significantly increased three days post-ICH but were reduced by FMT. (Figure 5C–5E).

FMT from mice treated with Ber led to a marked elevation in the Arg-1^+^Iba^+^/Iba^+^ cell ratio three days post-ICH in comparison with the ICH+FMT (Vehicle) mice (Figure 5F, 5G). Arg-1 and CD206 levels were also elevated in the ICH+FMT (Berberine) group in comparison with Vehicle-only treated animals (Figure 5H, 5I). These results demonstrate that FMT from Ber-treated mice can lower neuroinflammation through regulation of the microglial phenotype after ICH. It is thus apparent that the gut microbiota is key to the therapeutic effects of Ber on brain damage induced by ICH.

### FMT from Ber-treated ICH mice improved neurological function after ICH

FMT from ICH Ber-treated mice was performed to assess the role of the gut microbiota in Ber-mediated recovery from ICH injury. This involved the transplantation of fecal material from mice treated with Ber into ICH mice and a subsequent comparison of the neurobehavioral functioning of the ICH mice treated with Ber and those treated with vehicle only to determine the effects of FMT on ICH-induced brain injury. The neurobehavioral tests were conducted as described above. Both the FMT-treated and Vehicle-only treated groups scored the same on all three behavioral tests on the first day after ICH. However, on the second and third days, the ICH+FMT (Berberine) group showed significantly reduced positive scores on the cylinder test compared with the Vehicle-only group (Figure 5J). Similarly, mice in FMT + Ber-treatment group scored significantly higher in the forelimb placement test and wire-hanging test compared to the controls were markedly better than those of the controls (Figure 5K, 5L).

### FMT from Ber-treated ICH mice reduces intestinal permeability after ICH

**Figure 6:**
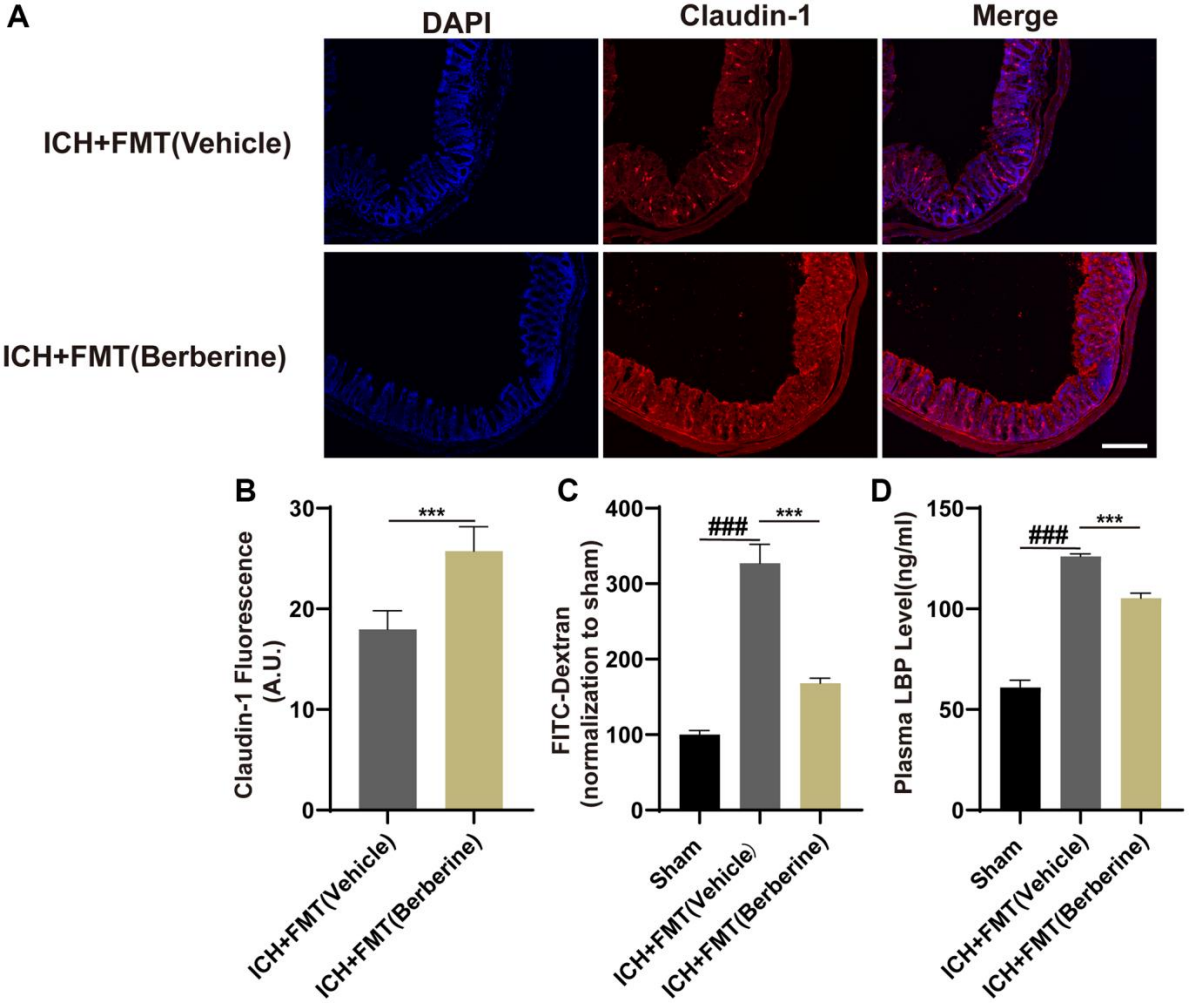
**Transplantation of fecal microbiome from Ber treatment ICH mice improves the gut function.** (**A**) The representative image of immunofluorescence staining of the tight junction protein Claudin-1 in ICH + FMT (Vehicle) and ICH + FMT (Ber) at day 3. (**B**) The mean of densities of Claudin-1 after ICH day 3. (*n* = 5 per group). (**C**) FMT treatment improved the intestinal permeability after ICH day 3. (*n* = 5 per group). (**D**) The level of LBP in plasma after FMT treatment after ICH day 3. (*n* = 5 per group). Data are expressed as the mean ± SEM. ^#^*P* < 0.05. ^##^*P* < 0.01. ^###^*P* < 0.001 sham vs. ICH + FMT (Vehicle) group. *P < 0.05. ^**^P < 0.01. ^***^P < 0.001 ICH + FMT (Vehicle) vs. ICH + FMT (Ber) group. Scale bar = 200 μm.

The effects of FMT from Ber-treated mice on intestinal barrier function after ICH were examined by assessing the levels of colonic tight junction proteins, plasma LBP concentrations, and intestinal permeability. The fluorescence intensity of Claudin-1 decreased after ICH in the ICH + FMT (Vehicle) mice but increased after treatment with FMT + Ber (Figure 6A, 6B). Furthermore, the levels of FD-4 in the plasma showed that FMT was able to reverse the damage caused by intestinal permeability by ICH (Figure 6C).

In addition, the plasma levels of LBP in the mice that received FMT were similar to those of the control mice and less than those of the ICH + Vehicle group (Figure 6D). Thus, it appears that intestinal integrity was restored after FMT from Ber-treated mice.

## DISCUSSION

Here, we examined the effects of Ber on neuroinflammation induced by ICH. We investigated the modulatory effects of Ber on microglial/macrophage phenotypes in the brain after ICH injury and whether Ber treatment promoted neurological recovery from ICH. We also demonstrated that Ber-mediated therapeutic effects were dependent on the gut microbiota and found that treatment with Ber could restore intestinal permeability and reduce LBP levels in the plasma.

It has been documented that both immune and neuroinflammatory responses play major roles in the secondary damage caused by ICH and that these responses significantly affect the prognosis of patients with ICH [26]. Microglia/macrophages are the immune cells of the brain. They are activated after ICH injury to promote inflammation in the brain. Activated cells show either pro- or anti-inflammatory phenotypes [27]. Inflammation-promoting microglial cells produce inflammatory cytokines, promoting neuroinflammation and aggravating the injury caused by ICH. In contrast, anti-inflammatory microglia produce cytokines and neurotrophic factors that lower inflammation and thus protect neurons [1, 27]. Earlier studies have suggested that modulation of the microglial phenotype from pro- to anti-inflammatory could protect against ICH-induced injury in animal models [6, 28]. However, these findings have not been extended into the clinical domain, and the prognosis of ICH patients remains poor. New therapeutic targets for injuries induced by ICH are thus necessary.

The gut microbiota has been implicated in neuroinflammation associated with several CNS diseases [29]. Chronic cerebral hypoperfusion in rats was found to adversely affect the microbiota, causing reduced levels of short-chain fatty acids and cognitive impairment, and these effects could be reversed by FMT [30]. In mice with TBI, the microbiota was found to reduce both neuroinflammation and promote neurogenesis [13]. Similarly, in SCI, where the homeostasis of the gut microbiome suffers severe injury, FMT was found to enhance both locomotory and intestinal function and reduce neuroinflammation [11, 12]. Furthermore, ICH produces gut dysbiosis, and in turn, the disrupted microbiome can aggravate neuroinflammation [15]. Nevertheless, the relationship between Ber and neuroinflammation induced by ICH is poorly understood, and exploring this relationship may provide a new direction for ICH treatment. In recent years, studies have reported an increase in intestinal barrier permeability after ICH [31], which can lead to microglia activation through inter-organ communication with intestinal-derived proinflammatory cytokine [32, 33]. In our study, we found that the expression of the tight junction proteins Occludin and ZO-1 was reduced after ICH, and that Ber treatment could ameliorate the disruption of the gut barrier. Our results suggest that Ber may reduce neuroinflammation by attenuating the disruption of the gut barrier.

Ber is a naturally occurring alkaloid that is widely used as an oral treatment for cardiovascular disease, type 2 diabetes, and hypertension [34, 35]. It has also been found to reduce inflammation in several acute and chronic diseases of the CNS. For instance, Ber treatment after ischemic stroke was found to reduce both neuroinflammation and neuronal apoptosis, promoting functional recovery [36]. In Alzheimer’s disease model animals, Ber could reduce amyloid deposition, assist in the clearance of tau protein accumulations, and promote recovery of function and neuronal protection [37]. Similarly, in a model of Parkinson’s disease, Ber was found to protect dopaminergic neurons, improving their viability as well as the overall motor function of the animal [38]. However, the precise mechanism underlying Ber action is not understood. Here, on the basis of the reported information on Ber effects in regulating neuroinflammation and microglial phenotypes in other diseases of the CNS, we hypothesized that Ber regulates neuroinflammation induced by ICH. Our results showed that Ber reduced inflammation after ICH by shifting the microglial phenotype from a pro-inflammatory state to an anti-inflammatory one, thereby reducing the expression of the pro-inflammatory microglial factors such as TNF-α, IL-1β, and CD16, and elevating the levels of the anti-inflammatory microglial factors such as CD206 and Arg-1, contributing to neurological recovery.

Ber is a widely prescribed traditional Chinese medicine that has anti-inflammatory effects. Ber is also known to have neuroprotective functions, although the precise mechanism is unclear. It has been reported that the gut microbiota is involved in Ber’s therapeutic effects [39]. Although it has also been shown to be effective in treating inflammation caused by hemorrhagic stroke, whether these effects are dependent on the microbiota is not known. Previous articles have reported that Ber can reduce the permeability of the blood-brain barrier [40]. While Ber may have a direct role in the CNS, these studies have not ruled out the influence of gut microbiota. In our study, we focused on the protective effect of gut microbiota, as there is considerable evidence suggesting that gut microbiota can influence the integrity of the blood-brain barrier. [40–42]. Here, we used broad-spectrum antibiotic administration to deplete the microbiota and observed that this depletion was able to block the anti-inflammatory effects of Ber after ICH. In our study, alpha diversity was mainly used to indicate the community composition of bacteria; β-diversity indicates the similarity between a community or individual sample and other samples [43]. ABx significantly lowered the diversity and abundance of the gut microbiota. In addition, FMT using intragastric administration of feces from mice treated with Ber was found to reduce inflammation. This was supported by the reductions in pro-inflammatory factor levels in the microglia and macrophages in the injured brain. In addition, FMT from Ber-treated animals was found to significantly improve the neurological functioning of mice after ICH. All these results indicate that Ber functions through the gut microbiota, although the precise mechanism, as well as the specific effects of microbiota components on ICH-induced injury, requires further investigation.

ICH may result in intestinal dysfunction in human patients as well as cause intestinal paralysis and increased permeability in animal models [15]. This may be the result of dysbiosis of the gut microbiota. Ber has been found to ameliorate damage to the intestinal barrier in diabetes and other disorders [18, 44]. Thus, we investigated whether Ber modulated intestinal function after ICH in mice. Ber treatment was shown to increase the levels of tight junction proteins in the epithelium and to promote recovery from barrier injury. Ber also reduced the plasma levels of LBP. FMT from Ber-treated mice also rescued intestinal barrier function, increasing the levels of tight junction proteins and reducing those of FD-4 and plasma LBP. These findings indicate that Ber promotes recovery from gut dysfunction induced by ICH and that the gut microbiota is key to this process, although the precise mechanism requires further investigation.

Although we have demonstrated that Ber reduces neuroinflammation and enhances outcomes after ICH and that this effect is dependent on the gut microbiota, the study has several limitations. First, the underlying mechanisms responsible for the changes in the microbiota after Ber treatment were not evaluated, and further investigation is necessary to determine the precise links between gut microbes and neuroinflammation. Second, we only used male mice in the study, and it is possible that female hormones may influence ICH outcomes. Third, it is known that age influences both the composition of the microbiome and the prognosis of many CNS-associated diseases. Finally, we focused on the brain-gut axis of Ber in reducing neuroinflammation, although Ber may directly play a role in neuroinflammation. The exact mechanism of Ber on microglial phenotype remains to be elucidated. The present study used young rather than old mice, and thus, further research is needed to explore this aspect in older animals.

## CONCLUSION

Our findings demonstrated that Ber reduces neuroinflammation through modulation of the microglial phenotype and promotes recovery from ICH and that this effect may depend on the gut microbiota. These results may offer new avenues for the prevention of ICH-induced secondary brain injury.

## Supplementary Materials

Supplementary Figure 1
