# Supplementary material for: Berberine mitigates intracerebral hemorrhage-induced neuroinflammation in a gut microbiota-dependent manner in mice
Source: Aging (Albany NY). 2023 Apr 7;15(7):2705–20. doi: 10.18632/aging.204642 (PMC10120891; doi:10.18632/aging.204642)
Supplement: Supplementary Figure 1 [file aging-15-204642-s001.pdf]

SUPPLEMENTARY FIGURE

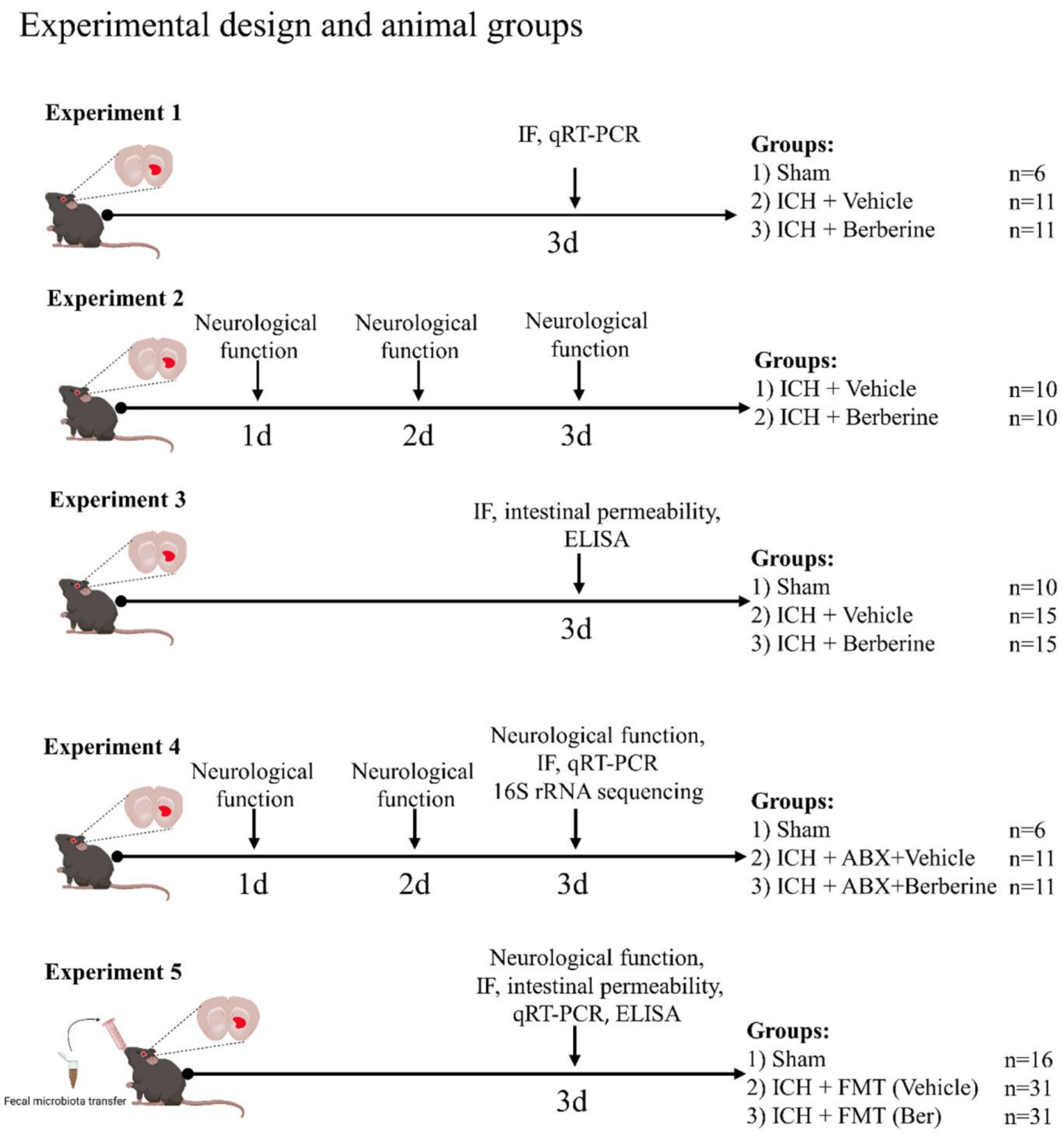

Supplementary Figure 1. The experimental design schematic, drug dosages, and animal groups (Part Figure was created with BioRender.com).
